# Prevalence and Factors Associated with a High Risk of Malnutrition among Older Adults in the East Coast of Peninsular Malaysia

**DOI:** 10.21315/mjms2024.31.6.15

**Published:** 2024-12-31

**Authors:** Asma Amaran, Nani Draman, Nur Suhaila Idris, Sakinah Harith

**Affiliations:** 1 Klinik Kesihatan Naka, Pekan Naka, Kuala Nerang, Kedah; 2 Department of Family Medicine, School of Medical Sciences, Universiti Sains Malaysia, Kelantan, Malaysia; 3 School of Nutrition and Dietetics, Faculty of Health Sciences, Universiti Sultan Zainal Abidin, Terengganu, Malaysia

**Keywords:** older persons, geriatric, high risk of malnutrition, malnutrition

## Abstract

**Background:**

Older people are more susceptible to malnutrition. Malnutrition is defined as imbalances and deficiencies of nutrients that result in diminished function. However, malnutrition identification through nutrition screening is not routinely performed at Malaysian health clinics or hospitals. Our study aimed to determine the proportion of older people at high risk of malnutrition and its associated factors.

**Methods:**

This was a cross-sectional study conducted among older persons aged ≥ 60 years, and the exclusion criteria were older persons with known cases of dementia or the inability to stand and have hand problems that limit the ability to hold the dynamometer. Sociodemographic data and anthropometry assessment were conducted. Malnutrition risk screening tool–hospital, modified Barthel Index and the Elderly Cognitive Assessment Questionnaire were used in this study. The data were analysed using descriptive statistics and multiple logistic regression.

**Results:**

A total of 200 older persons participated in the study, and the proportion of the high risk of malnutrition was 27 (13.5%). Poor handgrip strength odd ratio (OR) = 3.56, 95% confidence interval (CI) = 1.41, 8.98; *p* = 0.007) and living arrangements (OR = 4.6, 95% CI = 1.31, 16.1; *p* = 0.017) were significantly associated with a high risk of malnutrition in older persons.

**Conclusions:**

The proportion of older persons at high risk of malnutrition was low (13.5%). Poor handgrip strength and living arrangements are significant factors associated with a high risk of malnutrition among older persons. Nutrition screening can help identify the cause and other factors of malnutrition. The role of healthcare personnel should be emphasised in nutrition screening, as they are commonly the first point of contact for patients seeking medical advice.

## Introduction

Globally, the population is becoming older. The number of people in the globe who are ≥ 60 years of age was estimated to be 1 billion in 2020; this figure is expected to rise to 2.1 billion by 2050 due to population growth (1). In this age group, malnutrition is one of the main issues. A state of nutrition known as malnutrition is characterised by an excess of energy, protein or other nutrients that results in quantifiable negative consequences on body composition and a reduction in numerous activities (2). The proportion of undernourished elderly patients varies from 6% to 74.5% worldwide (3). According to data from the World Health Organisation (WHO), the prevalence of malnutrition in older adults ranged from 1.3% to 47.8% (4). Singapore, a wealthy nation in Asia, reported that 27.7% of senior citizens were at high risk of malnutrition (5).

According to the statistics from Malaysia’s National Health Morbidity Survey 2018, approximately 23.5% of this vulnerable population suffers from high risk malnutrition (6). In this susceptible and delicate population, malnutrition has a significant negative influence on health outcomes, such as longer hospital stays that result in functional impairment, a lower quality of life, a higher risk of infections, anaemia, electrolyte imbalances, lethargy and muscle atrophy (7–9). This may have a significant negative effect on healthcare costs and may result in financial, familial and economic strains (10). A state of malnutrition risk exists before malnutrition, allowing for intervention and prevention. Therefore, individuals who exhibit nutritional compromise before malnutrition development may be identified using nutritional risk screening.

The nutritional condition of elderly patients is affected by several variables, such as their physical, social and psychological conditions. The social factors contributing to malnutrition include inadequate intake, low appetite and social isolation (11, 12). Furthermore, malnutrition is linked to depressive disorders and chronic illnesses that affect appetite, as well as poor dental health that hinders the absorption of food (12). A weak handgrip strength reduces one’s capacity to prepare food, which in turn decreases nutrient intake (13). Malnutrition and cognitive function are also related. A study conducted on an older population in China using data spanning 7 years revealed that malnutrition and cognitive impairment are generally common and worsen with age (14).

Malnutrition is a well-established geriatric syndrome; thus, nutrition screening may be characterised as an early step to identify patients who are malnourished and at risk. This can be performed as part of a comprehensive geriatric assessment. For a more comprehensive intervention, nutrition screening must be implemented using practical, user-friendly and proven technology. Additionally, nutrition screening is necessary to promptly manage malnutrition in older adults to prevent worse health outcomes.

For use with older persons, numerous nutrition screening instruments are available. Malaysian hospitals and community organisations serving senior citizens have created and validated the malnutrition risk screening tool–hospital (MRST-H), the subjective global assessment and the global indicator of malnutrition (GIM) (15). These instruments are employed to evaluate undernourishment in older hospital patient cohorts (16, 17). Conversely, the mini nutritional assessment (MNA) is another nutritional screening tool that has been validated and is used in the community to identify older individuals who are malnourished or at risk of malnutrition (18).

Prior research on malnutrition in older adults was conducted in Malaysia. The prevalence of malnutrition in the community environment ranged from 17% to 52.6% for those at risk and from 4.3% to 13% for older people who were malnourished. In Malaysian society, the risk of malnutrition is present in 34%–43.1% and 56.9%–66.0% of male and female older individuals, respectively (19, 20). However, malnutrition is a slow-moving, progressive illness; thus, neither the affected individual nor a healthcare practitioner can readily recognise the signs and symptoms of malnutrition in older adults (21). Therefore, the purpose of this study was to ascertain at the primary care level the percentage of older individuals at high risk of malnutrition and the contributing factors.

## Methods

From June to December 2019, a cross-sectional study was conducted at a primary care clinic in a tertiary Hospital in Kelantan, Malaysia. All senior citizens aged ≥ 60 years were included in the convenience sampling. Senior citizens with dementia and are unable to stand or have hand difficulty in holding hand dynamometers were excluded. On the basis of a study conducted in Kajang, Selangor, the sample size was determined using software to calculate the power and sample size when comparing two means (22). A minimum of 174 samples were required. The estimated sample size, considering a 20% dropout rate, was a total of 218 patients.

### Research Tools and Materials

The questionnaire consisted of three parts: parts A, B and C. Part A consisted of the participants’ sociodemographic, functional assessment and elderly cognitive assessment questionnaire (ECAQ).

Functional assessment was performed using a valid and reliable Malay version of the modified Barthel Index (23). It measures the ability to complete 10 basic daily living activities: feeding, dressing, grooming, bathing, control of urinary bladder and bowel, transfer to bed, using the toilet, mobility and climbing stairs. The modification includes coding for help from 0 (unable) to 5 (independent) to increase sensitivity. The internal consistency reliability coefficient was 0.90 (24). A respondent was categorised as ‘functional independent’ in doing an activity if he or she could perform all 10 activities independently. A respondent was categorised as being a ‘functional dependent’ if he or she had a difficulty or needed help in performing one or more of the activities (23).

Cognitive assessment was performed using the Malay version of the ECAQ (25). It consists of three sections: memory (three items), orientation (six items) and memory recall (one item). It has a sensitivity of 85.3% and specificity of 91.5% (3). Respondents were categorised as having probable cognitive (score 0–4), borderline cognitive (score 5–6) or normal cognitive (score 7–10) function.

Part B includes nutritional screening. It was conducted using the Malay version of the MRST-H. MRST-H is a validated, simple and readily available nutrition screening tool developed for older persons in Malaysia (4). MRST-H showed 66.7% sensitivity, 96.2% specificity and 82.4% positive predictive value to GIM (4). MRST-H consists of five items, which provide financial status, self-feeding, unintentional weight loss (either < 5% weight loss in 1 month or > 10% in the past 6 months) and anthropometric measurements (mid-upper arm and calf circumference [MUAC and CC]), respectively. The total score ranges from 0 to 8. Respondents were categorised as having a low nutritional risk (MRTS-H < 2 score) and a high malnutrition risk (MRTS-H score ≥ 2).

Finally, part C is concerned with anthropometric measurements. Anthropometric measurements included height, weight, MUAC and CC. Height and weight were measured twice and the mean was calculated. Body mass index (BMI) was calculated using the BMI formula = [weight in kg/height in m^2^]. The BMI was categorised into four groups: underweight (< 18.5 kg/m^2^), normal weight (18.5 kg/m^2^–22.9 kg/m^2^), overweight (23 kg/m^2^–27.4 kg/m^2^) and obesity (≥ 27.5 kg/m^2^).

MUAC was measured using measuring tape at the midpoint between the tip of the shoulder and the tip of the elbow, with the participant standing and relaxing his arm to the side with the palm facing the thigh. CC was measured in the standing position. The largest CC was measured in centimetres, midway between the ankle and knee. All measurements were taken thrice and recorded to the nearest 0.1 cm, and the mean value was determined (11).

Handgrip strength was measured using a handgrip. It is a simple, non-invasive marker of upper extremity muscle strength, assessed using a hand dynamometer TKK 5101, Grip D with a maximum strength of 100 kg. In a standing position with the forearm at 90°–180° with the arm, subjects applied a few seconds of maximal hand gripping with alternating hands. A 10 s rest was given in between the measurements to minimise fatigue. The same researcher conducted all tests to control the inter-tester variability. The readings of the two trials from the right and left were recorded to the nearest 0.1 kg, and the mean of both hands was used in the subsequent analysis (26). In this study, the poor handgrip was set as < 27.0 and < 16.0 kg in men and women, respectively (26).

### Data Collection Procedure

Older patients aged > 60 years who fulfilled the inclusion and exclusion criteria were identified and invited to participate in the study. Consent was obtained, and a face-to-face interview to answer the questionnaires and assessment was performed by the investigator with a duration of 20 min–30 min. Clinical history and medical records were also used. Finally, height, weight, MUAC and CC were measured. The examination was completed using handgrip strength assessments. Patients were informed of the results. Patients with clinically significant malnutrition were given an appointment with a dietician for further evaluation. Patients with severe cognitive impairment based on the ECAQ were referred to a medical officer for further management. All patient answers were strictly confidential.

### Data Entry and Analysis

Data analyses were performed using International Business Machines Corporation Statistical Package for the Social Sciences (IBM SPSS) statistics version 26.0 (Armonk, New York). The numerical variables are expressed as mean and standard deviation (SD), and categorical variables are expressed as frequency and percentage. Variables with *p*-values < 0.25 in the simple logistic regression and are clinically significant with a high risk of malnutrition were included in the multiple logistic regression. The dependent variable is high risk of malnutrition. The forward and backward methods were used to predict variables associated with high risks of malnutrition. On the basis of the final model, the variables included in the model are assessed for their interactions.

## Results

A total of 218 older adults who met the eligibility requirements were enrolled; 200 of them completed the research, yielding a 91.7% response rate.

### Characteristics of Respondents

The age of respondents was 60 years–93 years, with a mean age of 68.4 (SD = 6.60). Among the respondents, 25 (92.6%) were Malays, 22 (81.5%) were married, 17 (64.0%) had no formal education, 22 (81.5%) were jobless, 11 (40.7%) were overweight and obese and 18 (66.6%) had a family income of more than RM1,000. Most of them were Malays. Table 1 summarises the specifics of the respondents’ medical and sociodemographic characteristics.

Using the MRST-H, the study’s 200 older respondents showed a low proportion of high risk malnutrition: 27 (13.5%).

### Factors Associated with High Risk of Malnutrition

Living conditions and handgrip strength were significantly associated with a high risk of malnutrition. According to Table 2, the probabilities of high risk malnutrition were 4.6 for living with a family member and 3.6 for having poor handgrip.

## Discussion

According to the MRST-H score in this study, the prevalence of high risk malnutrition among older adults in primary care settings was 13.5%, which is low. Nevertheless, a previous study using a similar technique found that the prevalence of high risk malnutrition was greater than that in our study (approximately 40%) among older patients with stroke in the outpatient clinics of three government hospitals in Peninsular Malaysia (27). This is because patients with stroke have a higher risk of malnourishment due to reduced mobility, dysphagia and cognitive impairment (28). According to a study conducted in the same area as ours in Kelantan, 34.5% of older individuals hospitalised in Hospital USM were malnourished. Compared with that in older persons who are hospitalised, the frequency of high risk malnutrition in the community is lower because hospitalised older adults have more comorbidities (29).

However, the results of our study showed that the prevalence was comparable with that of Japan, with 13% of the 130 older people being at risk for malnutrition (30). Nevertheless, the low prevalence in Japan compared with that in other Asian communities may be explained by the country’s technological advancements in health care. Similarly, among older people living in Norwegian communities, the prevalence of malnutrition was found to be low (7.1%) compared with modern nations with sophisticated infrastructure and high economic status (31).

An additional study conducted in a care facility in Malaysia revealed that 13% of the participants were considered malnourished and 37% were at risk of malnutrition (32). According to a recent study conducted in the Klang Valley among community People’s Housing Projects flats in Kuala Lumpur, 3.3% of residents were malnourished and 29.6% were at high risk of malnutrition (33). Because the study included 6045 participants and was conducted on a considerably larger scale, the prevalence of people at risk of malnutrition was found to be 27.6% in the Singaporean community (34). Compared with our analysis, all these investigations demonstrated a higher prevalence of a high risk of malnutrition because of differences in sample size, comorbidities, socioeconomic background and nutritional tool use.

Our study’s prevalence is much lower than that of other studies due to many factors, such as our respondent sample size was smaller (n = 200) compared with that of other studies (n = 488–3,977). Our respondents also had fewer comorbidities, as reported by Bellanti F et al., where the prevalence of malnutrition increased by age and comorbidities (29). Furthermore, our study was conducted in a general outpatient clinic that does not specifically cater to geriatric or specified clinics, such as stroke or geriatric clinics. Additionally, our study used the MRST-H for nutritional screening, whereas their studies used the MNA tool.

MNA is a nutritional assessment tool used at the community level. It can detect malnourished or those at risk of malnutrition, particularly in older patients aged ≥ 65 years (18). This MNA tool is widely used in research in many countries and was developed in Europe. However, the questionnaires and cutoff point for CC were different for our local population in Malaysia. Thus, in 2012, MRST-H was developed for local use in Malaysia to determine the high risk of malnutrition in older persons within the Malaysian population (15). It has been used to screen for malnutrition among older inpatients and outpatients in hospital settings in Malaysia (15, 19), and this study was conducted in a primary care setting.

Our respondents were also found to have a higher prevalence of overweight and obesity. Malnutrition is also associated with obesity. The diagnosis of malnutrition in individuals with obesity can be challenging. To identify malnutrition in obese patients, it is necessary to assess both their nutrition and nutritional status (35). The most common methods for dietary assessment are 24 h/48 h dietary recalls. Identifying malnutrition also involves anthropometric measurements, blood biochemical tests and physical parameters.

In patients with obesity, despite excessive energy consumption, there is often a shortage of individual microelements. Deficiencies or imbalances in essential micronutrients can significantly affect daily performance, intellectual and emotional well-being and physical state of the body (36). Nutrient deficiencies in individuals with obesity may be partly due to the overconsumption of high-calorie foods with low nutrient density (37). Astrup and Bugel suggested that, in addition to recommending healthy eating and increased physical activity for individuals who are overweight and with obesity, supplementation with specific micronutrients or vitamins should be considered for those at the highest risk of deficiencies (38).

Poor handgrip strength and living arrangements were two important characteristics found in this study related to the high risk of malnutrition in older adults. It is commonly recognised that sarcopenia and fragility are related to handgrip strength (13). Local research on older Malaysian residents in nursing homes revealed a link between malnourishment and weak handgrip strength (39). Conversely, ferritin levels and handgrip strength were associated in that study. Both muscle tiredness and activity will decrease in the presence of a ferritin deficiency (39). Nevertheless, ferritin was not assessed in our participants because biochemical measurements were not part of the nutritional screening process. Comorbidities in the older population, reduced physical activity and ageing itself result in muscular atrophy and changes in muscle structure and muscle fibre composition leading to a decline in muscle strength, subsequently leading to poor handgrip. In older people, handgrip strength reflecting hand function is important for meal preparation and food intake.

Living arrangements, specifically living in a family, were revealed to be another related factor associated with a greater risk of malnutrition in this study. According to a study conducted in India by Tyagi, Kapoor and Kapoor (2008), 259 women who stayed with their families were underweight compared with those who were in a nursing home. However, in that study, low and high BMI were used to define the dietary status. Because women ate erratically and did not exercise much, they were overweight and obese (40). WHO states that obesity, overweight and underweight are included in the malnutrition spectrum (41). The majority of those who stayed with family were unemployed and financially reliant, according to the sociodemographic statistics. Additionally, they are probably old and fragile, which prompts them to live with family members.

This study has certain limitations. Because of its cross-sectional design, non-response bias was possible. Malays comprised most of the research’s respondents, and it was a small-scale study. Therefore, the prevalence observed in this study cannot be indicative of a larger population and should not be extrapolated to other ethnic groups or rural locations. Additionally, this study did not investigate numerous other important predictors of malnutrition, including polypharmacy, nutritional knowledge, decreased appetite, bad dentition and depression. An additional consideration is poor dentition.

It is our recommendation that community-based nutrition education programmes are implemented to encourage healthy eating habits and ageing in this susceptible population. Dietitians should be consulted for specialised nutritional interventions if individuals are at nutritional risk. Nutrition interventions should also include psychosocial and physical components. Additionally, there may be a predominance of psychological issues related to coping with the illness of the older population and these individuals may withdraw from society, leading to social isolation, depression and decreased appetite and activity, all of which can exacerbate physical issues like fatigue and malnourishment. Future studies should consider additional variables such as polypharmacy, dietary awareness, decreased appetite, bad dentition and depression.

## Conclusions

In summary, 13.5% of older patients attending a semi-government primary care clinic were at high risk of malnutrition. According to the study findings, living conditions and weak handgrip strength are linked to an increased risk of malnutrition. Treatment and referral should be given to older patients at high risk of malnutrition. To identify and avoid malnutrition, which inadvertently increases the quality of life, nutrition screening is essential. Furthermore, in community and healthcare settings, proper dietary treatments may be one of the most important tactics for controlling and preventing health issues in the older population.

## Figures and Tables

**Table 1:** Sociodemographic and medical characteristics of respondents (n = 200)

Variable	Total n (%)	High risk malnutrition n (%)	Normal nutrition n (%)
Age (years)	68.4 (6.60)^a^	67.5 (6.30)^a^	68.5 (6.65)^a^

Gender			
Male	106 (53.0)	13 (48.1)	93 (53.8)
Female	94 (47.0)	14 (51.9)	80 (46.2)

Ethnicity			
Malay	180 (90.0)	25 (92.6)	155 (89.6)
Non-Malay	20 (10.0)	2 (7.4)	18 (10.4)

Marital status			
Married	161 (80.5)	22 (81.5)	139 (80.3)
Single/Widow	39 (19.5)	5 (7.5)	34 (19.7)

Living arrangement			
Alone/with a partner	64 (32.0)	3 (11.1)	61 (35.3)
With family	136 (68.0)	24 (88.9)	112 (64.7)

Educational level			
Formal education	38 (19.0)	10 (37.0)	28 (16.2)
No formal education	162 (81.0)	17 (63.0)	145 (83.8)

Employment			
Employed	30 (15.0)	5 (18.5)	25 (14.5)
Unemployed	170 (85.0)	22 (81.5)	148 (85.5)

Family income (RM)			
≥ 1000	124(62.0)	18(66.6)	106(61.3)
< 1000	76 (38.0)	9 (33.4)	67(38.7)

Disease status Diabetes mellitus			
Yes	106 (53.0)	13 (48.1)	93 (53.8)
No	94 (47.0)	14 (51.9)	80 (46.2)

Hypertension			
Yes	155 (77.5)	18 (66.7)	137 (79.2)
No	45 (22.5)	9 (33.3)	36 (20.8)

Hyperlipidaemia			
Yes	163 (81.5)	21 (77.8)	142 (82.1)
No	37 (18.5)	6 (22.2)	31 (17.9)

Modified Barthel’s Index			
Independence	173 (86.5)	21 (77.8)	152 (87.9)
Dependence	27 (13.5)	6 (22.2)	21 (12.1)

Body mass index (kg/m^2^)			
Underweight	9 (4.5)	5 (18.5)	4 (2.3)
Normal weight	46 (23.0)	11 (40.7)	35 (20.2)
Overweight and obese	145 (72.5)	11 (40.7)	134 (77.4)

ECAQ score	8.9 (1.61)^a^	8.4 (1.55)^a^	9.0 (1.61)^a^

Handgrip male (kg)	27.5 (7.19)^a^	23.9 (7.08)^a^	28.0 (7.11)^a^
Handgrip female(kg)	16.3 (5.62) a	12.5 (4.49)^a^	16.9 (5.56)^a^

Handgrip			
Good	68 (34.4)	26 (35.2)	42 (31.8)
Poor	132 (65.6)	48 (64.8)	90 (68.2)

Notes:

a mean (SD)

**Table 2:** Associated factors for high risk of malnutrition in the older person using simple logistic regression and multiple logistic regression analysis

Variable	Crude OR^a^ (95%CI)	*p*-value	Adjusted OR^b^ (95%CI)	*p*-value
Age	0.98 (0.92, 1.04)	0.484		
ECAQ score	0.842 (0.68, 1.04)	0.104*		

Gender				
Male	1			
Female	1.25 (0.56, 2.82) 0.588			

Ethnicity				
Non-Malay	1			
Malay	1.45 (0.32, 6.64)	0.631		

Marital status				
Married	1			
Single	0.93 (0.33, 2.63)	0.890		

Educational level				
Formal education	1			
No formal education	3.05 (1.26, 7.34)	0.013*		

Living arrangement				
Alone/with a partner	1		1	
With children/family	4.36 (1.26, 15.06)	0.020*	4.6 (1.31, 16.1)	0.017

Employment				
Unemployed	1			
Employed	1.35 (0.47, 3.88)	0.583		

Family income (RM)				
≥ 1000	1			
< 1000	0.79 (0.34, 1.86)	0.592		

Disease status				
Diabetes mellitus				
Yes	1			
No	1.25 (0.56, 2.82)	0.588		
Hypertension				
Yes	1			
No	1.90 (0.79, 4.59)	0.520		
Hyperlipidaemia				
Yes	1			
No	1.31 (0.49, 3.51)	0.593		

Handgrip				
Good	1		1	
Poor	3.40 (1.37, 8.46)	0.008*	3.56 (1.41, 8.98)	0.007

Modified Barthel Index				
Independence	1			
Dependence	2.07 (0.75, 5.71)	0.161*		

Notes:

a (SLR) simple logistic regression;

* *p-*value of ≤0.25 are considered significant in SLR, multiple logistic regression.

Backward LR was applied. Multicollinearity does not exist and no significant interaction is present. The model assessment was met. Hosmer-Lemeshow test showed *p* = 0.595, classification table (overall correctly classified percentage) 86.5% and area under the ROC curve was 71.0%.
